# Evaluation of Herbal Mouthwashes Containing Zataria Multiflora Boiss, Frankincense and Combination Therapy on Patients with Gingivitis: A Double-Blind, Randomized, Controlled, Clinical Trial

**DOI:** 10.31661/gmj.v8i0.1366

**Published:** 2019-07-15

**Authors:** Zoleikha Khoshbakht, Ehsan Khashabi, Laleh khodaie, Mohammadali Torbati, Farzaneh Lotfipour, Hamed Hamishehkar

**Affiliations:** ^1^Department of Phytopharmacy, Faculty of Traditional Medicine, Tabriz University of Medical Sciences, Tabriz, Iran; ^2^Department of Periodontics, Faculty of Dentistry, Urmia University of Medical Sciences, Urmia, Iran; ^3^Medical Philosophy and History Research Center, Tabriz University of Medical Sciences, Tabriz, Iran; ^4^Department of Food Science and Technology, Faculty of Nutrition, Tabriz University of Medical Sciences, Tabriz, Iran; ^5^Food and Drug Safety Research Center, Faculty of Pharmacy, Tabriz University of Medical Sciences, Tabriz, Iran; ^6^Drug Applied Research Center, Tabriz University of Medical Sciences, Tabriz, Iran

**Keywords:** Frankincense, *Zataria multiflora* Boiss, Mouthwashes, Chlorhexidine

## Abstract

**Background:**

Dental plaques as adhesive microbial aggregates on tooth surfaces are considered the first stage of tooth decay as well as gingivitis. Accordingly, the effect of different antimicrobial mouthwashes on removing dental plaques and preventing their formation has been evaluated in various studies. This study aimed to evaluate the efficacy of herbal mouthwashes containing hydro-alcoholic extract of Zataria multiflora (ZM), Frankincense (FR), and a combination of both (ZM+FR) and compare it with chlorhexidine (CHX) mouthwash in subjects with gingivitis.

**Materials and Methods:**

In this randomized, controlled, clinical trial a total of 140 patients with gingivitis were divided into four groups including CHX (control group), ZM, FR, and ZM+FR groups. Plaque index (PI), gingival index (GI), and gingival bleeding index (GBI) were measured in days 1, 14, and 21.

**Results:**

All three herbal types of mouthwash significantly improved plaque, gingivitis, and gingival bleeding throughout days 14 to 21 (P<0.001). There was no difference between herbal mouthwash with CHX groups. CHX mouthwash showed the most side effects (54.3%), while ZM mouthwash showed the least side effects and the highest consumer satisfaction (5.7% and 94%, respectively).

**Conclusion:**

All of the herbal mouthwashes can be good candidates for controlling gingivitis. Comparing with CHX mouthwash, herbal mouthwashes have lower side effects and negligible alcohol content. Among the herbal mouthwashes, ZM outperforms FR and FR+ZM due to its lower side effects and higher levels of patients’ satisfaction.

## Introduction

Gingivitis is a mild and reversible inflammation of the gingival tissue that manifests with symptoms such as erythema, edema, and spontaneous or provoked bleeding [1]. Gingivitis, if left untreated, can progress to periodontitis that is known as one of the main causes of tooth loss [1-4]. It is one of the most prevalent diseases of humankind, especially in developing countries [1, 3]. Mechanical plaque removal is the most effective way to prevent and treat periodontal plaque-related diseases [5]. However, antiseptic mouthwashes have been suggested to overcome its limitations in accessing hard-to-reach spaces and enhance plaque removal process [6]. Chlorhexidine (CHX) is an effective and low-cost mouthwash with a broad antimicrobial activity that has been used routinely in recent thirty years and is considered to be a gold standard mouthwash [7]. The tendency to attach bacterial cell wall and negatively charged tooth surfaces is a prominent characteristic of CHX that enables it to be retained in the oral cavity for 8-12 hours. This property also prevents its absorption from the digestive system and limits its systemic side effects [8, 9]. Despite the above advantages, CHX side effects such as tooth staining, supra-gingival calculus formation, and unpleasant taste in mouth are considered to be obstacles to its acceptance and routine use by the patients [10]. To find an optimal mouthwash, complementary medicine sources have suggested interesting alternatives that mainly concentrate on herbal medicine. The Iranian traditional medicine practitioners recognized the importance of oral health, such that in traditional medicine sources, separate chapters have been devoted to oral and dental diseases [11]. In traditional Iranian pharmacopeia called “*Qarabadine*,” more than 50 medicinal plants in forms of gargle, mouthwash, powder, and oral douches have been recommended to treat oral and dental problems [12, 13]. In these formulations, plants such as Salvadora persica, *Aloe vera*, *Acacia arabica*, *Buniump ersicum*, *Rosa damascena*, and *Rhus coriaria* were used either alone or in combination with other materials [2].*Zataria multiflora* Boiss (ZM) belongs to the Lamiaceae family and is native to central and southwest Asia. It is used widely in alternative medicine in various pharmaceutical forms including syrup, tablet, oral drop, soft capsule, and vaginal cream. ZM has been widely studied, and its antiseptic, antifungal, antioxidant, spasmolytic, anti-nociceptive, and antimicrobial properties have been reported. Antimicrobial properties of ZM have been the most researched aspect of the plant [14]. ZM has many similarities to *Thymus vulgaris* in terms of phytochemistry and pharmacological properties [15]. It possesses several systemic and topical indications having been mentioned in complementary herbal medicines, and many of them have been confirmed in recent researches [16-18]. In the section devoted to oral and dental problems in the complementary medicine, ZM has been recommended to be used in forms of powder and mouthwash to eliminate oral infections and halitosis and relieve dental pain and gingival wounds [12]. Frankincense (FR) is a fragrant gum resin derived from Boswellia tree belonging to the Burseraceae family [19]. Despite being not native to Iran, this plant is known to traditional medicine therapists. It has also been used in forms of mouthwash and powder for preventing gingival bleeding and halitosis and healing gingival wounds [12]. Many researchers have identified and studied the active ingredients of the mentioned mouthwashes and suggested their applications in oral and dental diseases as well as central nervous system diseases [20, 21]. This study aimed to evaluate the clinical efficacy of ZM, FR, and ZM+FR mouthwashes and compare it with CHX mouthwash for improving gingival health considering related indices and patient’s satisfaction.

## Materials and Methods

### Patients

**Figure 1:**
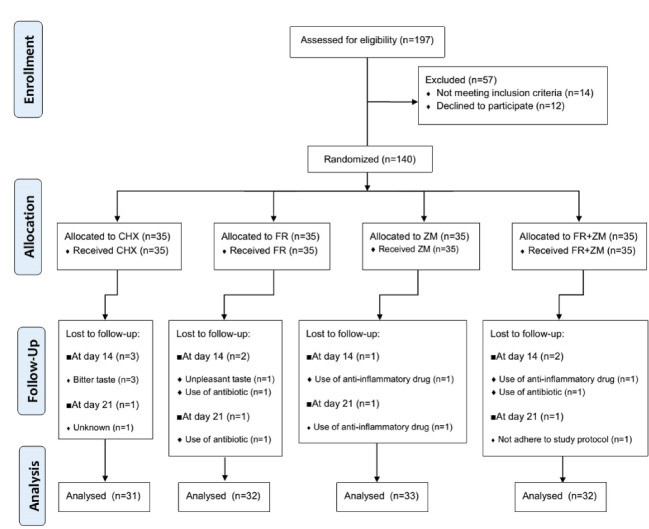
CONSORT flowchart of study.

This study was a double-blind, randomized, clinical trial that conducted on 140 eligible volunteers referring to the Department of Periodontics, Faculty of Dentistry, Urmia. The sampling was started after obtaining permission from the Research Ethics Committee of Tabriz University of Medical Sciences (ethical code: REC.1396.161) and registered in the study in Iranian Registry Clinical Trials (RCT code: IRCT2017072435263N1). Flowchart of the present study is shown in Figure-1. The inclusion criteria were the plaque index (PI) higher than 1.9, gingival index (GI) higher than 1, no consumption of any drugs (i.e., antibiotics and steroidal anti-inflammatory drugs) one month before and/or during the study, literacy, and access to phone, and at least 17 years old and having at least 20 teeth. The exclusion criteria were metabolic diseases (e.g., diabetes mellitus), use of medicines that cause gingival hyperplasia (e.g., cyclosporine, phenytoin, and calcium channel blockers), smoking, use of any mouthwash since four weeks before the study, sensitivity to plant materials, pregnancy and breastfeeding, undergoing dental procedures outside the scope of the study, and being under orthodontic treatments.

### Sample Size Calculation

The sample size was calculated using the R software ver. 3.0.2. According to the previous studies [22, 23] and power of 80%, and also considering four experimental groups, 35 participants were needed for each group. Also, the probable dropout rate was considered 10%.

### Plants Collection and Preparation of Mouthwashes

Dried aerial parts of ZM Boiss and oleo-gum-resin of FR were provided from a medicinal plants market (Barg-e-Sabz Herbal Drug Store, Iran) and authenticated by a botanist. The hydro-alcoholic extracts of aerial parts of ZM and oleo-gum-resin of FR were prepared by maceration using ethanol and water (70/30). Rotary evaporator at 45˚c was used for drying hydro-alcoholic extracts. The amount of extracts was 35% and 48% for ZM and FR, respectively. The obtained extracts were standardized according to pharmacopeia monographs. Thymol was determined as the main active constituent of ZM using Gas Chromatography-Mass Spectrometry (GC-MS), and 11-keto-β-boswellic acid was determined as the active constituent of FR extract using High-Performance Liquid Chromatog raphy (HPLC). The prepared herbal mouthwashes and CHX (Behsa Co, Iran) were filled in similar dark containers with well-fit cap, in such a way that it was not possible to detect the type of mouthwash from its appearance. Oral and written hygiene instructions including how to use a toothbrush, dental floss, and mouthwash were given to the volunteers. They also received a package containing one mouthwash and medium toothbrush (Signal, Unilever France HPC Industries, France), a dental floss (Mina waxed dental floss, Mina Co., Iran), and a 10cc cup. They were asked to brush their teeth at least three times a day, use dental floss, rinse 10cc of mouthwash for 1 minute twice a day (after breakfast and before going to bed), and avoid eating or drinking until one hour later. To evaluate consumption, patients were asked to bring mouthwash bottle at their next visit. Professional prophylaxis was performed on all groups’ participants at day 0.

### Randomization, Blinding, and Allocation

Mouthwashes were labeled with A, B, C, and D (A: CHX, B: FR, C: ZM, and D: ZM+FR). Researcher, examiner, patient, and statistician were not aware of the contents of the bottles. The pharmacist was the only person being aware of the contents of the bottles. Patient’s assignment to study groups was done using the random numbers table (Permuted Block Randomization). In total, 140 patients were divided into four groups of 35 members. The control group received CHX 0.2% mouthwash while the other three groups received one of the herbal mouthwashes, i.e., ZM extract, FR extract, and a combination of both (ZM+FR). After obtaining informed consent, the profile of the patients was recorded.

### Outcomes Measurement

According to the experimental model of gingivitis and its treatment described by Leo *et al*. [24, 25], the study period was determined 21 days, and measurement was carried out three times on days 1, 14, and 21 from the beginning of the study. Variables were PI, GI, and gingival bleeding index (GBI) [26, 27]. The quantitative and qualitative details of the indexes have been described in Table-1. Patients’ satisfaction was determined with the numerical scale ranging from 0-2 (0: strongly dissatisfied, 1: not adequately satisfied, and 2: strongly satisfied). At first visit, patients were asked to brush and use dental floss in the correct way, and then the study indices were examined and recorded. A single calibrated examiner did all the examinations. The calibration was performed on 15 subjects possessing all of the inclusion criteria. Examinations were repeated at different intervals, and the results were analyzed using the kappa statistic (ҡ). The efficient intra-examiner reliability was 0.74, 0.83, and 0.82 for PI, GI, and GBI, respectively, indicating a good agreement in the examination. The allocated mouthwash letter, visiting timetable, and directions for using mouthwash were written on the mouthwash bottles label. Every week with a telephone call, patients were reminded about using mouthwash and the next visit time. At every visit, patients were asked about mouthwash usage. If mouthwash failed to be used for two days or more, the patient was excluded from the study (Figure-1).

### Statistical Analysis

Data were analyzed using SPSS software (version 16, Chicago, SPSS Inc., United States) Qualitative data analysis was conducted using the nonparametric Wilcoxon signed-rank test, and quantitative data analysis was done with ANOVA and paired t-test. The mean and standard deviation were measured for the quantitative variable, and the percentage frequency was measured for the qualitative variables. The P-value<0.05 was considered the significance level.

## Results

In total, 140 patients with a mean age of 28.9 years (min: 17, max: 49) participated in this study. Most of the participants were female (F: 104, M: 36). Participants were allocated to four groups. The results of three visits on days 1, 14, and 21 were recorded and compared.

### PI

All the studied groups showed a significant decrease in PI on days 14 and 21 (P<0.001), as demonstrated in Table-2. Comparison between the studied groups showed no significant difference in PI reduction either between the control group and herbal mouthwashes or among the herbal mouthwashes under scrutiny on days 14 and 21 (Table-2).

### GI

All four types of mouthwashes were significantly effective in reducing GI on days 14 and 21 (P<0.001). In Table-2, the potency of different mouthwashes in reducing gingival inflammation has been compared. The potency of ZM mouthwash in the reduction of the mild inflammation of gingival tissue was the most even more than that of CHX mouthwash. The GI in the ZM group reduced 42.9% and 80% after 14 and 21 days, respectively.

### GBI

Study of gingival GBI data in the studied groups showed that the efficacy of all mouthwashes to decrease the GBI during days 14 to 21 was significant (P<0.001, Table-2).

### Adverse Effects

Comparison of probable side effects of herbal and CHX mouthwashes showed that 54.3% of the patients received CHX experienced various side effects, such as burning sensation, mucositis, and bitter taste. Among the herbal mouthwashes, ZM had the least side effects (5.7%) followed by FR and ZM+FR mouthwashes with 13.4% and 14.3%, respectively. The most reported side effect for FR and ZM+FR mouthwashes was an unpleasant taste that was absent in ZM mouthwash.

### Patients Satisfaction

To determine the patients’ satisfaction with mouthwashes, the numerical scale was used. The most satisfaction was for ZM mouthwash (94%) followed by FR, ZM+FR, and CHX mouthwashes with 78%, 72%, and 52%, respectively ( Table-3).

## Discussion

This study showed significant improvement of gingivitis after using ZM, FR, and ZM+FR mouthwashes as well as CHX mouthwash. These effects can be due to their antimicrobial and anti-inflammatory properties. To date, various studies have been conducted on the

anti-inflammatory and antimicrobial effects of ZM. Shahin *et al*. showed that ZM mouthwash was more effective than Myrtus communis in decreasing the recovery time and the average time of removing aphthous stomatitis [28]. In a similar study, ZM mouthwash, which was used twice a day for four weeks was considerably more effective than placebo in removing [29]. In another study, the antimicrobial effects of ZM extract on reduction of the count numbers of *Streptococcus mutans*, *Enterococcus faecalis*, and *Candida albicans* colonies were shown [27]. ZM essential oil consists of two phenolic and non-phenolic components [14]. Thymol and Carvacrol as the main antimicrobial ingredients are present in a phenolic component with various ratios, and p-cymene is the main ingredient of the non-phenolic component [18]. It has been demonstrated that the antimicrobial effect of ZM extract is due to flavonoids, more polar thermolabile, thermo-stable phenols, and also rosmarinic acid [30]. It has been shown that polar section of ZM extract has the same antimicrobial effects as its essential oil on growth inhibition of *S. aureus*, *S. marcescens*, *S. flexneri*, *Bacillus* subtilis, *E. faecalis*, *E. coli* and *Klebsiella pneumoniae* [30]. Although not statistically significant, ZM showed higher anti-inflammatory properties compared to all other mouthwashes group. Thymol is likely to be responsible for this effect because it has been shown that it can inhibit the release of elastase as an inflammation cause for neutrophils stimulated by chemotactic peptides. It is believed that calcium channels occupation and inactivation by strongly hydrophobic terpenoid phenols such as Thymol and Carvacrol found in ZM essential oil is responsible for changing calcium level in intracellular fluid and reducing elastase, leading to ZM anti-inflammatory effects [31]. FR oleo-gum-resin is a product of Boswellia Serrata Roxb [32]. It is also known as Indian frankincense or Indian olibanum and is abundantly found in northern and central parts of Indian under the name of Shallaki [32]. FR is composed of resin, polysaccharides, and essential oil with different content ratios depending on the harvest time and geographic location of the plant [33]. The resin portion consists of monoterpenes (alpha-thujone), diterpenes, triterpenes, pentacyclic triterpene acids, and tetracyclic triterpene acids. Boswellic acids are among the main components of resin section [20]. Antibacterial and antifungal effects of oleo-gum-resin of FR has been proven [34]. Among different boswellic acids found in the resin section, 3-O-Acetyl-11-Keto-β-Boswellic Acid (AKBA) is mainly responsible for anti-inflammatory effects by inhibiting 5-lipoxygenase enzyme [32]. AKBA is also the main inhibitor of gram-positive bacteria [35]. The results of antimicrobial studies on oral pathogens showed that AKBA is the most effective ingredient against *S. mutans* and *E. faecalis* [36]. FR has been used in the management of inflammatory bowel diseases and rheumatoid arthritis; thus, it is a good candidate for controlling oral inflammatory condition [37]. Nevertheless, to the best of our knowledge, no clinical study has ever evaluated the efficacy and side effects of oral products of FR. More notably, FR has also astringent properties that may be responsible for its anti-inflammatory effects [38]. As alcohol content of mouthwashes is considered a drawback, many new types of mouthwash have been widely produced using alcohol-free formulations. Ethanol is mainly used as a co-solvent in herbal mouthwashes such as Listerine [39]. Despite many pieces of evidence on the efficacy of mouthwashes in inhibiting oral bacteria, there have been serious concerns about their alcohol content [39]. Most concerns are associated with the direct effects of alcohol on the upper digestive tract. Alcohol is responsible for upper gastrointestinal tract damage and increased cellular proliferation causing leukoplakia and dysplasia and/or cancer [40-42, 3]. Moreover, ethanol metabolic conversion both by body’s cell and by oral microflora can result in acetaldehyde concentration [43, 44]. Alcohol can also be the source of severe allergic reactions [45, 46]. This clinical study had some limitations mentioned in the following. Firstly, the follow-up period of the study was relatively short, because more time seems to be required to find out the side effects of herbal mouthwashes including discoloration of the teeth. Secondly, although the study protocol was explained to the patients at the beginning of the study, and they were given oral and written explanations about the proper way of brushing, flossing, and using mouthwash, and they were asked to return the mouthwash container on days 14 and 21, we were not completely sure about the precision of consumption by the patients. Thirdly, it seems that more accurate results will be obtained if microbial culture is contemporary performed with a clinical study. The results of this study are economically valuable. According to the results, there is no requirement for simultaneous use of both extracts to achieve acceptable results, and ZM extracts are preferable with regard to their low percentage of side effects and high patients’ satisfaction.

## Conclusion

The products used in this study are deemed to be superior to traditional mouthwashes containing CHX due to their negligible alcohol content. Our results showed no significant difference between ZM, FR, and ZM+FR. It can be rationally concluded that all three types of mouthwashes are suitable for gingivitis treatment; however, ZM mouthwash is a better choice as it had greater patient acceptance and lower complications such as tooth staining and bitter taste.

## Acknowledgment

This study was a part of the Ph.D. thesis of in traditional medicine [Zoleikha Khoshbakht]. This study was supported by the grant number 5/D/517567 and funded by the Tabriz University Medical Sciences. Also, we would like to thank the Urmia University Medical Sciences for providing the necessary support facilities. The authors would like to thank all those who supported us and participated in this study.

## Conflict of Interest

There are no conflicts of interest.

**Table 1:** The Qualitative and Quantitative Details of PI, GI, and GBI

**Index**	**Details**	
	**Qualitative**	**Quantitative**
**PI**	Patients are asked to hold a plaque disclosing tablet in the mouth.	In this method, the surface of each tooth is divided into six parts, and then the PI is calculated by dividing the number of colored surfaces by the total number of available surfaces.
**GI**	-	Healthy gums=0 Mild inflammation (brief change of color, brief edema, and absence of bleeding on probing)=1 Moderate inflammation (redness, edema, gum reflex, and bleeding on probing)=2 Severe inflammation (redness, edema, ulcer, and spontaneous bleeding)=3
**GBI**	Walking stroke is performed by moving the dental probe on gingival tissue, and after 30 seconds we will observe bleeding.	No-bleeding (the healthy appearance of gum and pulp)=0 Mild bleeding (change in color and edema)=1 Severe bleeding (frequent edema, change in the color, scar, and spontaneous bleeding)=3

**PI:** plaque index, **GI:** gingival index, **GBI:** gingival bleeding index

**Table 2:** Comparison of the Outcomes of the PI, GI, and GBI between Studied Groups

**Variables**		**CHX** **(n=31)**	**FR** **(n=32)**	**ZM** **(n=33)**	**FR+ZM** **(n=32)**	**P-value**
**PI, mean** **±SEM**	day 1	82.54±3.134	82.79±3.378	87.71±2.172	92.13±2.601	-
	day 14	52.80±3.164	54.76±2.910	57.54±2.792	63.58±2.571	<0.001
	day 21	35.37±2.771	37.43±3.204	33.65±2.399	92.13±3.274	<0.001
GI, n(%)*	day 1	25(80.65)^1^ 5(16.12)^2^ 1(3.22)^3^	24(75)^1^ 8(25)^2^	28(84.84)^1^ 5(15.15)^2^	24(75)^1^ 7(21.87)^2^ 1(3.13)^3^	-
	day 14	14(45.16)^0^ 16(51.61)^1^ 1(3.23)^2^	18(56.25)^0^ 13(40.62)^1^ 1(3.13)^2^	18(54.55)^0^ 15(45.45)^1^	16(50)^0^ 14(43.75)^1^ 2(6.25)^2^	<0.001
	day 21	24(77.41)^0^ 7(22.59)^1^	28(87.5)^0^ 4(12.5)^1^	30(90.90)^0^ 3(9.1)^1^	26(81.25)^0^ 6(18.75)^1^	<0.001
GBI, n(%)**	day 1	26(83.87)^1^ 4(12.90)^2^ 1(3.23)^3^	26(81.25)^1^ 6(18.75)^2^	30(90.9)^1^ 3(9.09)^2^	26(81.25)^1^ 5(15.63)^2^ 1(3.12)^3^	-
	day 14	16(51.62)^0^ 14(45.16)^1^ 1(3.22)^2^	19(59.38)^0^ 12(37.5)^1^ 1(3.12)^2^	19(57.57)^0^ 14(42.42)^1^	15(46.88)^0^ 15(46.88)^1^ 1(3.12)^2^ 1(3.12)^3^	<0.001
	day 21	25(80.65)^0^ 6(19.35)^1^	28(87.5)^0^ 4(12.5)^1^	33(100)^0^	27(84.38)^0^ 5(15.62)^1^	<0.001

**PI:** Plaque index; **GI:** Gingival index; **GBI:** Gingival bleeding index; **CHX:** Chlorhexidine; FR: Frankincense; **ZM:** Zataria multiflora; *Scale number of GI (healthy gum=0, mild inflammation=1, moderate inflammation=2, severe inflammations=3); **Scale number of gingival bleeding index (no-bleeding=0, mild bleeding=1, moderate bleeding=2, severe bleeding=3).

**Table 3:** Patient’s Satisfaction of Studied Groups

**Satisfaction level**	**CHX(n=31)** **n (%)**	**FR (n=32)** **n (%)**	**ZM (n=33)** **n (%)**	**ZM+FR (n=32)** **n (%)**
**Satisfied**	16 (51.6)	26 (81.25)	30 (90.91)	22 (68.75)
**Relatively satisfied**	11 (35.49)	5 (15.63)	3 (9.09)	8 (25)
**Strongly dissatisfied**	4 (12.9)	1 (3.12)	0 (0)	2 (6.25)

**CHX:** Chlorhexidine; **FR:** Frankincense; **ZM:** Zataria multiflora
